# Contribution of actin filaments and microtubules to cell elongation and alignment depends on the grating depth of microgratings

**DOI:** 10.1186/s12951-016-0187-8

**Published:** 2016-04-29

**Authors:** Kyunghee Lee, Ee Hyun Kim, Naeun Oh, Nguyen Anh Tuan, Nam Ho Bae, Seok Jae Lee, Kyoung G. Lee, Chi-Yong Eom, Evelyn K. Yim, Sungsu Park

**Affiliations:** Mechanobiology Institute (MBI), National University of Singapore, Singapore, 117411 Singapore; Department of Chemistry and Nano Sciences (BK21 plus), Ewha Womans University, Seoul, 120-750 South Korea; Department of Nano Bio Research, National Nanofab Center (NNFC), Daejeon, 305-806 South Korea; Seoul Center, Korea Basic Science Institute, Seoul, 136-713 South Korea; School of Mechanical Engineering, Sungkyunkwan University, Suwon, 440-746 South Korea; Department of Chemical Engineering, University of Waterloo, 200 University Avenue West, Waterloo, ON N2L 3G1 Canada

**Keywords:** Microtubules, Actin microfilaments, RPE-1, Microgratings, FN-line pattern

## Abstract

**Background:**

It has been reported that both chemical and physical surface patterns influence cellular behaviors, such as cell alignment and elongation. However, it still remains unclear how actin filament and microtubules (MTs) differentially respond to these patterns.

**Results:**

We examined the effects of chemical and physical patterns on cell elongation and alignment by observing actin filament and MTs of retinal pigment epithelium**-**1(RPE-1) cells, which were cultured on either fibronectin (FN)-line pattern (line width and spacing: 1 μm) or FN-coated 1 μm gratings with two different depths (0.35 or 1 μm). On the surface with either FN-line pattern or micrograting structure, the cell aspect ratios were at least two times higher than those on the surface with no pattern. Cell elongation on the gratings depended on the depth of the gratings. Cell elongation and alignment on both FN-line pattern and 1 μm gratings with 0.35 μm depth were perturbed either by inhibition of actin polymerization or MT depletion, while cell elongation and alignment on 1 μm gratings with 1 μm depth were perturbed only by MT depletion.

**Conclusions:**

Our results suggest that the contribution of actin filaments and MTs to the elongation and alignment of epithelial cells on microgratings depends on the groove depth of these gratings.

**Electronic supplementary material:**

The online version of this article (doi:10.1186/s12951-016-0187-8) contains supplementary material, which is available to authorized users.

## Background

Cells respond to chemical and physiological cues of various extracellular environments in vitro or in vivo. Cells integrate these various cues and generate appropriate cellular responses, such as alignment, migration, and elongation [1, 2]. Chemical cues are mediated by a specific integrin-ligand binding [3]. On the other hand, physical cues are generated by various topographic structures, such as gratings (anisotropic grooves and ridges), cliffs, pillars, and islands [4]. Only anisotropic topographic structures can induce different types of cells to align and elongate along the direction of axis. This phenomenon is called contact guidance which has been marked especially when the grooves or ridges are narrower than cells on the microgrooves [5–7]. It was hypothesized that contact guidance is a consequence of a cell’s ability to spread in the direction of higher rigidity, which is the length of the anisotropic structures [8, 9].

In previous studies, there have been significant differences in the cytoskeletal organization of cells located between topographic and flat surfaces [6, 10, 11]. When cells are cultured on topographic surfaces, actin filaments [10, 11] and microtubules (MTs) [6] align along the grooves and ridges, but these cytoskeletons do not show any preferential orientations on a flat surface. With alignment, actin filaments and MTs also play an important role in regulating cell length [12, 13] and cell spreading [14–16]. The cortical tension is mediated by actin and myosin. This tension plays a pivotal role in regulating cell shape and spreading [17]. The dynamics of MTs also influences the regulation of cell length and cell shape in fission yeast [18]. However, it has not yet to be clearly understood how cytoskeletons get differentially organized in the presence of different surfaces of topographical structures. For example, cell alignment and elongation are dependent on aspect ratio (depth/width) of anisotropic structures [8, 9], but it still remains unclear how actin filaments and microtubules (MTs) differentially respond to anisotropic structure with different aspect ratios.

In this study, we used a live-cell imaging technique to meticulously observe the alignment and elongation of non-transformed human retinal pigment epithelial (RPE-1), which are cultured on chemically or physically patterned anisotropic surfaces. Most of cancer cell lines are aneuploid conditions, which are caused by various mechanisms, including impaired microtubule dynamics [19]. Since we wanted to see the role of MTs on contact guidance, we chose RPE-1, which is one of the few cell lines that can maintain a diploid karyotype over an extended culture period. Using RPE-1, it has been determined how chemical patterns affect the shape and division of cells [17, 20]. In this study, RPE-1 cells were cultured in different substrates: tissue culture polystyrene (TCPS), TCPS printed with fibronectin (FN) lines (line width and spacing: 1 μm), and 1 μm polydimethylsiloxane (PDMS) gratings (0.35 or 1 μm depth) coated with FN. The aspect ratios (length/width) and alignment of cells on these substrates were subsequently investigated to determine how FN geometry and anisotropic surface topography affected contact guidance behaviors such as cell alignment and elongation. In addition, the actin filament and microtubule inhibitors, cytochalasin D (CD) and nocodazole (Noc), respectively, were used to investigate the roles of actin filaments and MTs on the behaviors.

## Results and discussion

### RPE-1 cells align and elongate along the direction of FN line or microgratings

**Fig. 1:**
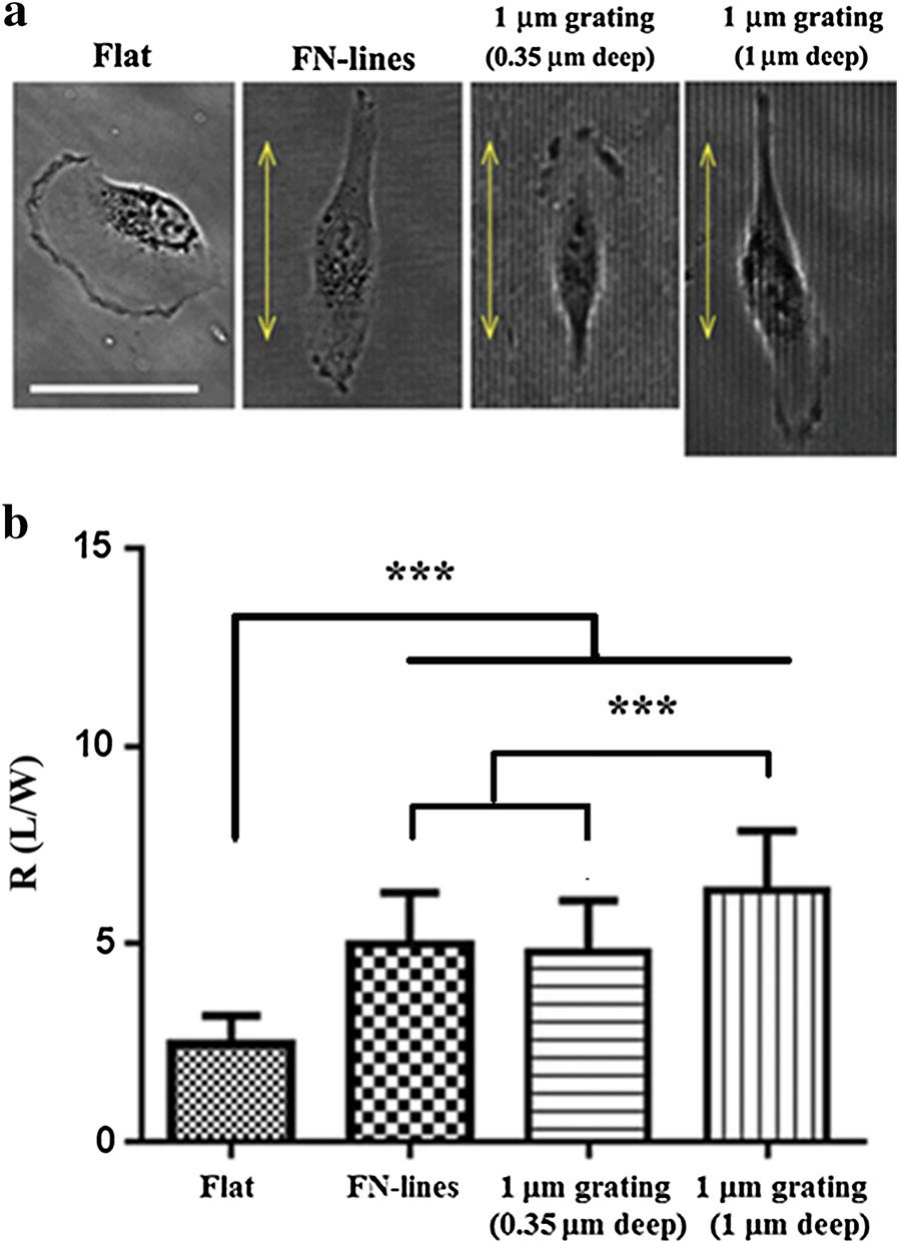
Cell alignment and elongation on different substrates. **a** Phase contrast images of cells on four different surfaces. *Bar* 50 μm. The *yellow arrow* indicates the long axis of either FN-lines or each micrograting. **b** Average aspect ratios (R) of cells on flat (n = 153), FN-lines (n = 141), 1 μm gratings (0.35 μm deep) (n = 111), 1 μm gratings (1 μm deep) (n = 123). *n* number of cells. ***P < 0.001. Data were analyzed using one-way ANOVA and a Bonferroni post hoc test. *Error bar* denotes the standard deviation of the mean

Cells on a flat surface of TCPS randomly orientated, most of cells on FN-lines of TCPS and 1 μm microgratings (0.35 and 1 μm deep) well aligned along the long axis (a yellow arrow) of all the patterns (Fig. 1a). When cell alignment on the patterns was determined by measuring angle differences between the major axis of cells and the long axis of FN-line or each micrograting, it was usually less than 15° (Table 1) that are deemed to be aligned according to a previous report [9].

**Table 1:** Effect of different substrates on cell alignment

Substrate	FN-lines^a^ (1 µm line)	1 µm grating^b^ (0.35 µm deep)	1 µm grating^c^ (1 µm deep)
Cell alignment (°)^d^	10.7 ± 7.7 (n = 106)^e^	8.4 ± 7.4 (n = 103)	3.8 ± 3.0 (n = 133)

*n* cell number

^a^FN-lines: TCPS microcontact-printed with FN: 1 µm line and spacing

^b^1 μm grating (0.35 μm deep): PDMS gratings (1 µm groove, 1 µm ridge, 0.35 µm deep) coated with FN

^c^1 μm grating (1 μm deep): PDMS gratings (1 µm groove, 1 µm ridge, 1 µm deep) coated with FN. *n*: number of cells

^d^Cell alignment is defined as the angular difference between the major axis of cells and the long axis of FN-line or each micrograting. It was measured at 12 h after seeding onto each substrate

For cell length analysis, the cell aspect ratio (R) was regarded as parameter at a fixed time point. R was defined as a ratio of cell length (L) to cell width (W). R values indicate that cells on FN-lines and 1 µm gratings (0.35 and 1 µm deep) were significantly more elongated than those on the flat surface of TCPS (Fig. 1b; Table 3). Cells on 1 µm gratings (1 µm depth) displayed the highest R values (Fig. 1b; Table 3) among the cells on the patterns (FN-lines and 1 µm gratings (0.35 and 1 μm deep). Wong et al. [9] reported that cell elongations on microngratings (1, 2 and 10 μm) decreased on wider gratings. Crouch et al. [8] reported a similar trend. It was also reported that compared to cells on microgratings, those on 100 nm gratings were less aligned to the direction of the grating axis [8]. Due to the reason, we chose 1 and 2 μm gratings. Cells on 2 µm gratings (2 µm depth) were slightly longer than those on 1 µn grating (1 µm depth) (Additional file 1: Figure S2), which is in accordance with the previous report [9]. However, cytoskeleton images of cells on 2 µm gratings were of poor quality because the gratings caused diffraction of light. Thus, we used 2 µm gratings only for cell alignment and elongation studies that do not require high resolution images. Since FN-lines were prepared by selectively coating TCPS surface with FN, they were considered as gratings with zero aspect ratio. The results (Fig. 1b; Table 3) indicate that cell elongation was dependent on grating aspect ratio (depth/width). Similar reports were found elsewhere [8, 9]. The cytoskeleton of cells on 1 µm gratings (1 µm depth) would have to be bended at a higher degree in order to conform to the grooves, compared to that of cells on either 1 µm gratings (0.35 µm depth) or FN-lines. Based on this explanation, cells on the deep gratings are less likely to spread in the lateral direction than those on both shallow gratings (0.35 µm depth) and FN-lines. Further studies on filopodium and lamellapodium formation on gratings are needed to support the explanation.

### Cell alignment and elongation on 1 µm gratings with 1 µm depth are not interfered by depletion of actin filaments

**Fig. 2:**
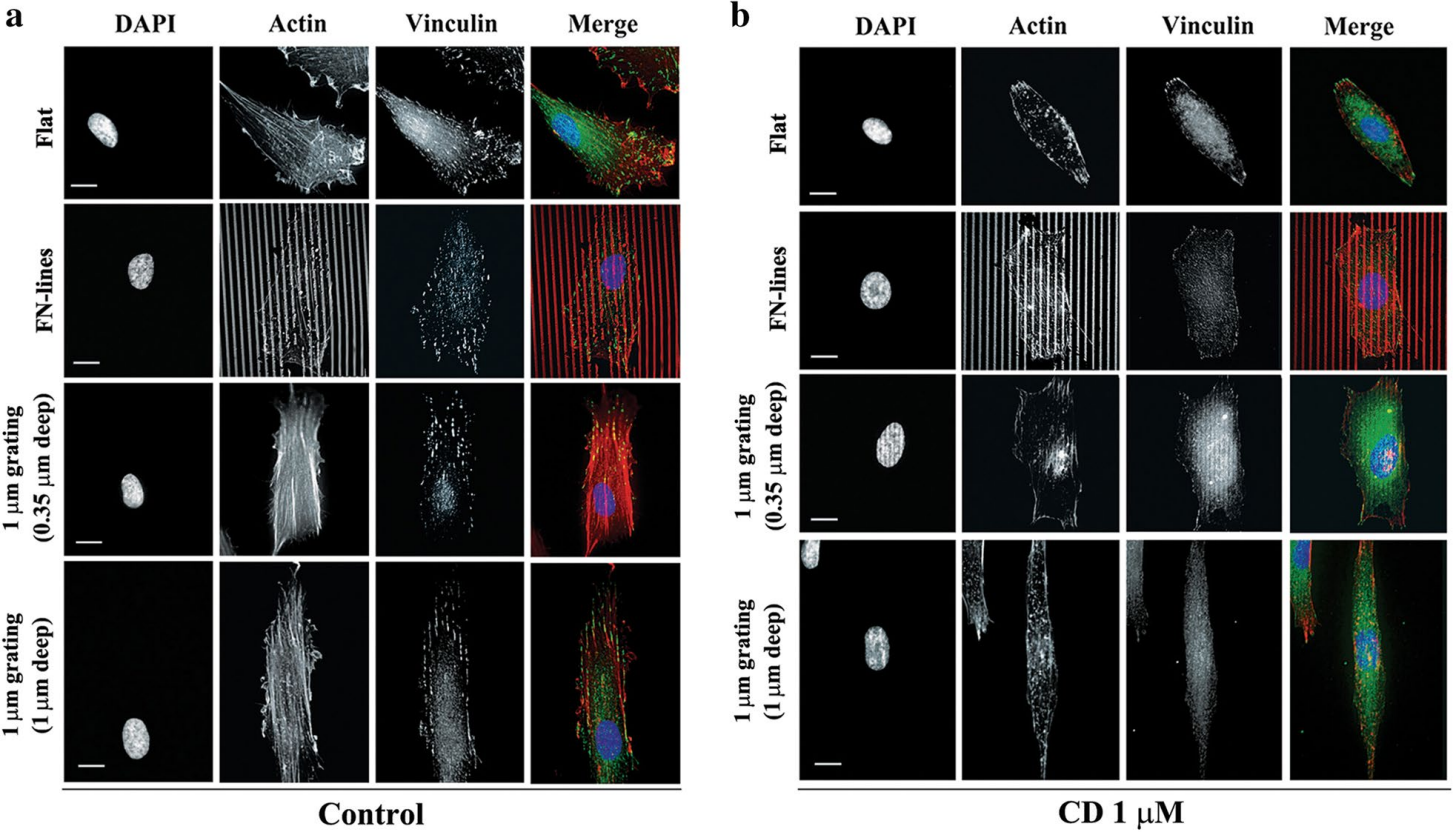
Effect of CD on FAs and actin filaments. Fluorescence images of nucleus (*Blue*), actin filaments (*Red*), and vinculin (*Green*) of cells on flat, FN-lines, and 1 μm gratings (0.35 or 1 μm deep) in the absence (**a**) or presence (**b**) of CD at 1 μm. *Bar* 10 μm

To understand how cells control their orientation and shape when exposed to anisotropic surfaces induced either by chemical or topographical effects, we seeded RPE-1 cells onto four different substrates (Flat, FN-lines, 1 µm gratings with 0.35 or 1 µm depth) in the absence or presence of CD, an inhibitor of actin filament polymerization, before staining. In the absence of CD, both actin filaments and vinculin, a focal adhesions (FAs) marker, were randomly orientated on the flat surface, while both molecules were aligned along FN-lines or along the direction of microgratings (Fig. 2a; Additional file 1: Figure S3). This indicates that focal adhesions (FAs) and actin filament assembly are involved in determining cell alignment and elongation in response to anisotropy of chemical and topographic patterns. When Cy3.5-FN had to be used to visualize the printed line of FN, we employed excitation and emission wavelengths of 557 and 578 nm, respectively. These wavelengths overlapped with those (576/589 nm) of tetramethyl rhodamine B isothiocyanate (TRITC)-phalloidin, which were used for actin staining. Thus, the images of actin filaments were blurred on FN lines.

**Fig. 3:**
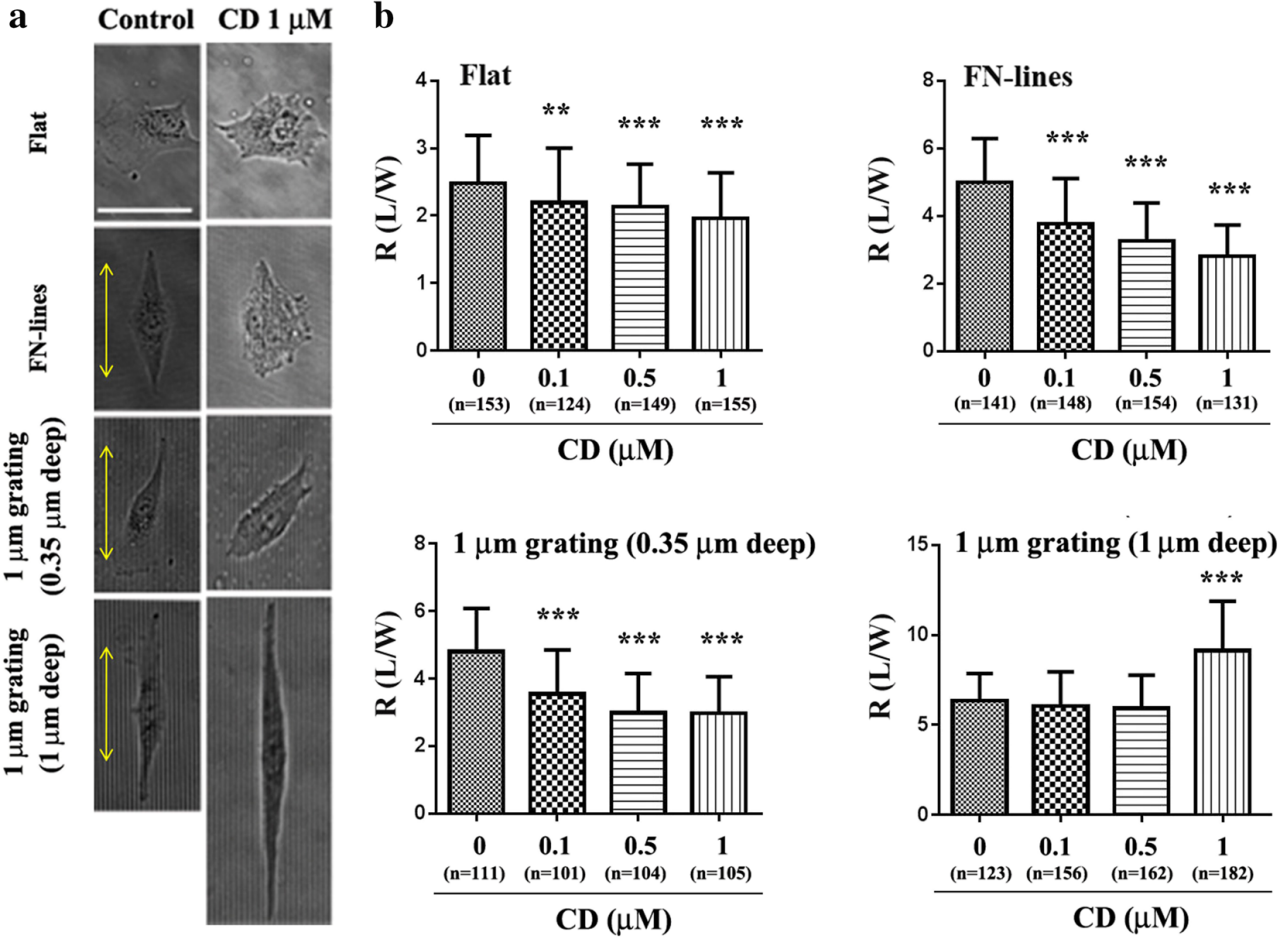
Effect of CD on cell elongation on different substrates. **a** Phase contrast images of cells on flat, FN-lines, and 1 μm gratings (0.35 or 1 μm deep) with/without CD treatment. A *yellow arrow* the long axis of the FN line pattern or each micrograting. *Bar* 50 μm. **b** Average cell aspect ratio (R) of cells on flat, FN-lines, or 1 μm gratings (0.35 or 1 μm deep) with/without CD treatment. *n* number of cells. **P < 0.01, and ***P < 0.001. Data were analyzed using one-way ANOVA and a Bonferroni post hoc test. *Error bar* denotes the standard deviation of the mean

In the presence of 1 µm CD, stress fibers and vinculin formed by actin polymerization were either significantly decreased or completely vanished from the cells on all the substrates (Fig. 2b), indicating that FAs were inhibited by the treatment. Cells on all the substrates were treated with 2 µm CD but many of them did not spread well (data not shown), which was reported elsewhere [6, 21, 22]. The CD-treated cells (Figs. 2b, 3a) on all the substrates except for cells on 1 µm grating (1 µm deep) looked shorter than untreated cells on their respective substrates. The observations are further supported by the cell aspect ratio (R) values (Fig. 3b) showing that CD treatment also caused a decrease in R values of cells on all the substrates except for R values of cells on 1 µm deep gratings. Interestingly, R values of cells on the 1 µm deep gratings were not significantly changed at very low concentrations of CD (0.1 and 0.5 µm) (Tables 2, 3) but were significantly increased by CD at 1 µm. This is further supported by the cell alignment data (Table 2). R values of cells on the 2 µm grating (2 µm depth) were increased by CD at 1 µm as well (Additional file 1: Figure S2). These results showed that contact guidance occurred in these deep gratings in the presence of CD. This suggests that focal adhesion and microfilament alignment is not a prerequisite for contact guidance in the deep gratings.

**Table 2:** Effect of drug treatments on cell alignment of different substrates

Drug treatment		Cell alignment (°)^a^		
Drug^b^	Concentration (µm)	FN-lines (1 µm line)	1 µm grating (0.35 µm deep)	1 µm grating (1 µm deep)
None		10.7 ± 7.7 (n = 106)	8.4 ± 7.4 (n = 103)	3.8 ± 3.0 (n = 133)
CD	0.1	10.0 ± 8.6 (n = 112)	8.7 ± 8.4 (n = 100)	3.9 ± 3.0 (n = 109)
	0.5	20.2 ± 16.5 (n = 108)	8.5 ± 7.5 (n = 104)	3.8 ± 3.0 (n = 104)
	1	28.2 ± 17.2 (n = 106)	17.4 ± 14 (n = 104)	2.6 ± 1.9 (n = 101)
Noc	0.04	21.7 ± 15.6 (n = 103)	18.4 ± 17.9 (n = 110)	6.8 ± 4.8 (n = 103)
	1	25.6 ± 16.3 (n = 110)	23.5 ± 19.4 (n = 108)	24.0 ± 18.3 (n = 104)

^a^Cell alignment was measured at 12 h after seeding onto each substrate with and without CD or Noc treatment

^b^Cells on each substrate (FN-lines, the gratings) were treated with either CD (0.1–1 μm) or Noc (0.04 or 1 μm) right after cell seeding

**Table 3:** Effect of drug treatments on cell aspect ratio (R) of different substrates

Drug treatment		Cell aspect ratio (R)^a^			
Drug	Concentration (µm)	Flat^b^	FN-lines (1 µm line)	1 µm grating (0.35 µm deep)	1 µm grating (1 µm deep)
None		2.5 ± 0.7 (n = 153)	5.0 ± 1.3 (n = 141)	4.8 ± 1.3 (n = 111)	6.3 ± 1.5 (n = 123)
CD	0.1	2.2 ± 0.8 (n = 124)	3.8 ± 1.3 (n = 148)	3.5 ± 1.3 (n = 101)	6.1 ± 1.9 (n = 156)
	0.5	2.1 ± 0.6 (n = 149)	3.3 ± 1.1 (n = 154)	3.0 ± 1.2 (n = 104)	5.9 ± 1.8 (n = 162)
	1	2.0 ± 0.7 (n = 155)	2.8 ± 0.9 (n = 131)	3.0 ± 1.1 (n = 105)	9.1 ± 2.8 (n = 182)
Noc	0.04	1.7 ± 0.5 (n = 149)	1.8 ± 0.6 (n = 102)	3.5 ± 1.0 (n = 123)	5.9 ± 1.3 (n = 112)
	1	1.5 ± 0.4 (n = 112)	1.5 ± 0.4 (n = 102)	1.8 ± 2.1 (n = 102)	1.7 ± 0.4 (n = 110)

^a^R was determined by measuring cell length (L) and cell width (W) at 12 h after seeding. L is defined as the length of the long axis of the cell, while W is defined as the maximum width of a cell axis that is perpendicular to the long axis

^b^Flat surface: TCPS coated with FN

**Fig. 4:**
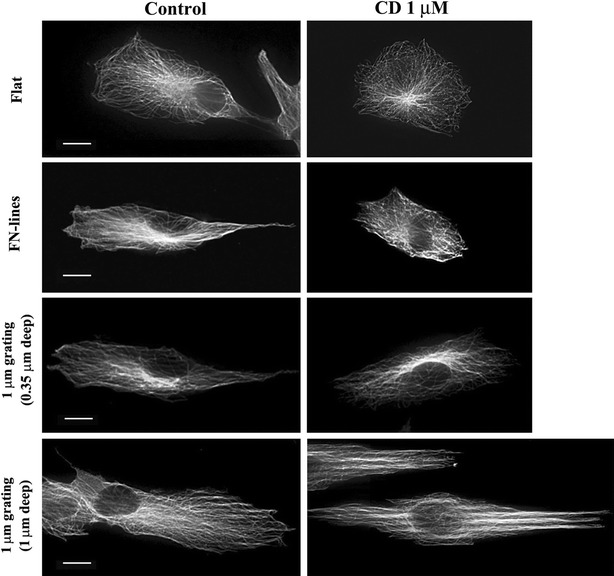
Effect of 1 µm CD on MTs on different substrates. MTs images of cells on flat, FN-lines or 1 μm gratings (0.35 or 1 μm deep) with/without CD treatment. After cells were treated with/without 1 μm CD for 16 h, they were fixed and stained with α-tubulin antibody for observing microtubules. *Bar* 10 μm

The use of CD at 1 µm concentration caused an increase in the length of cells on 1 µm deep gratings in spite of inhibition of actin and FA. To explain this phenomenon, we observed MTs, another major component of the cytoskeletons, in the absence or presence of CD at 1 µm. In the absence of CD, MTs of cells on a flat surface did not display any preferential orientation, while the MTs of cells on FN-lines and both types of microgratings aligned with the direction of anisotropic pattern of each substrate. Moreover, the MTs of cells on FN-lines and both types of microgratings were also axially elongated (Fig. 4). In particular, MTs on 1 µm deep gratings were denser and more likely to align along the axis of the grating than those on other substrates (Fig. 4). The orientation of MTs of cells on flat, FN-lines and 0.35 µm deep microgratings became disturbed when the MTs were treatment with 1 µm of CD (Fig. 4). It is known that initial orientations of MTs from the centrosomes are random but MT trajectories are aligned with targeted FAs by actin filaments as MTs grow [21]. It is thus expected that when actin filament assembly is inhibited, MTs are not able to be guided toward FA and maintain their initial orientations, which is relatively random compared to MTs in cells with intact actin filament assembly. This may explain why the CD-treated cells were less elongated compared to the untreated cells (Fig. 3) and their microtubules were not aligned to the grating axis. Based on our observations (Figs. 3, 4) and the previous finding [21], it is suggested that actin filaments play an important role in control cell shape by guiding the rigid cytoskeleton polymer MTs toward FAs in the shallow gratings and FN-lines.

Our results (Fig. 3) indicate that cells on 1 µm gratings (1 µm depth) were elongated when they were treated with CD. Compared to MTs that were not treated with CD on any gratings, the MTs were more condensed and aligned along to the ridges of 1 µm deep gratings when they were treated with 1 µm of CD (Fig. 4; Additional file 1: Figure S4). This hinted that unlike MTs on the shallow gratings those on the deep gratings were not able to be randomly orientated. These results suggest that the deep groove substratum compensates for the actin filament deficiency by fully enforcing condensation and elongation on aligned microtubules.

### Cell alignments on 1 µm deep gratings are interfered only by depletion of MT

**Fig. 5:**
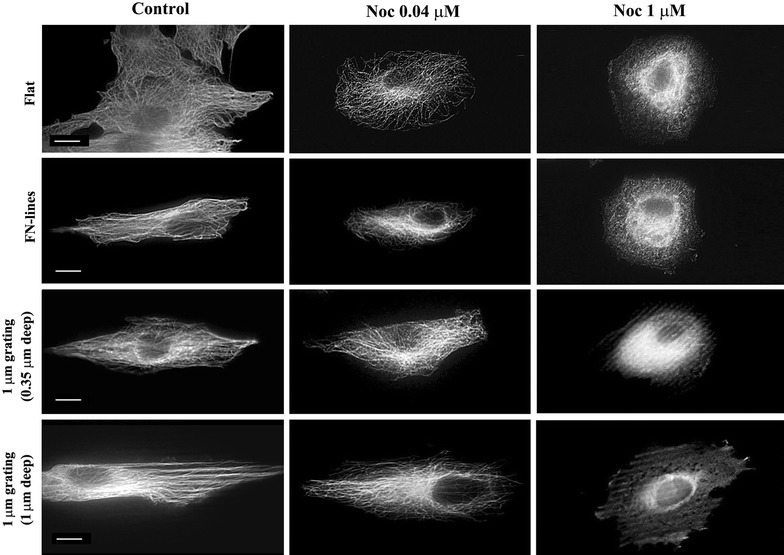
Effect of Noc on MT intensity and alignment of cells on different substrates. MTs Images of cells on flat, FN-lines or 1 μm gratings (0.35 or 1 μm deep) with/without Noc treatments. After cells were treated with/without Noc treatment (0.04 or 1 μm) for 16 h, they were fixed and stained with α-tubulin antibody for observing microtubules. *Bar* 10 μm

**Fig. 6:**
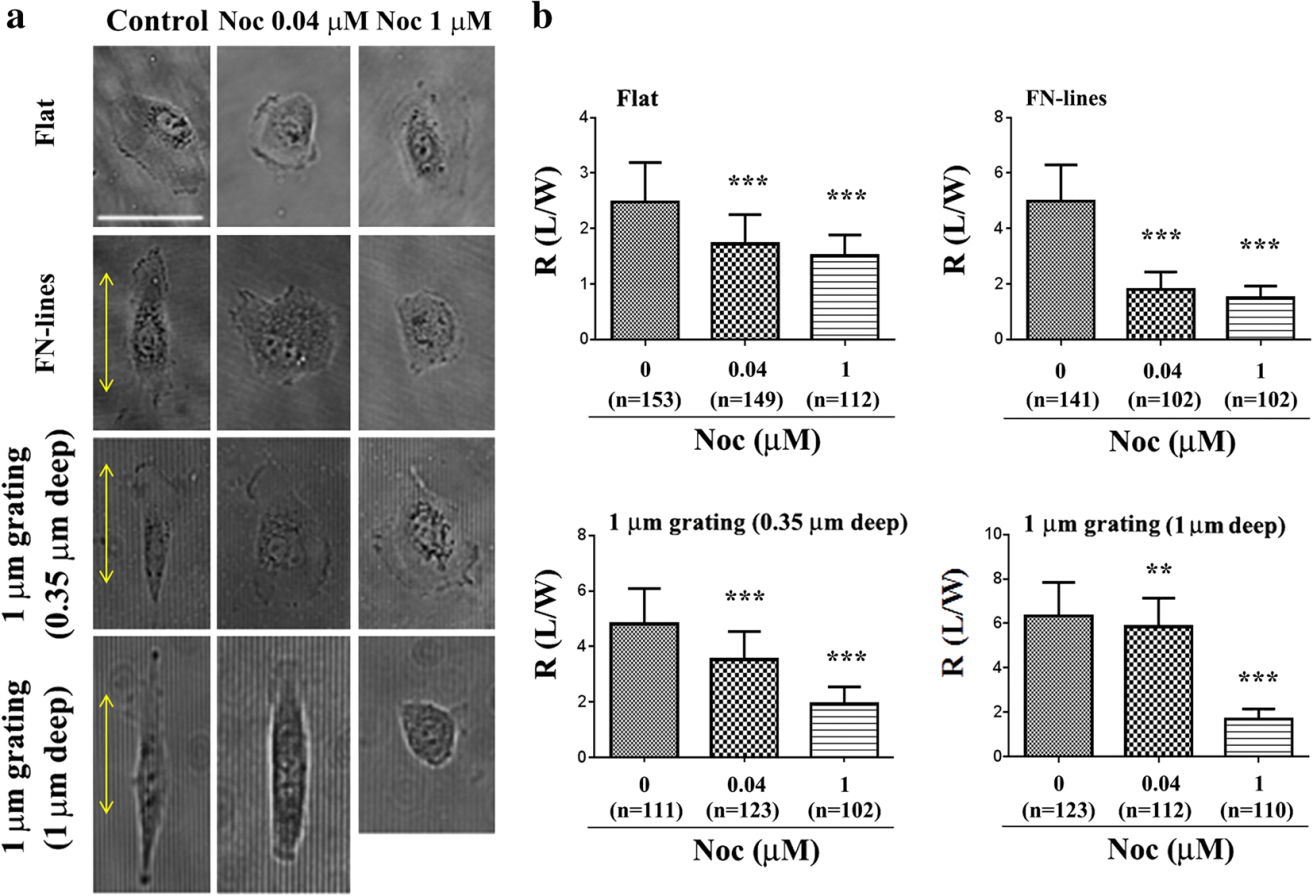
Effect of different concentrations of Noc on elongation of cells on different substrates. **a** Phase contrast images of cells on flat, FN-lines or 1 μm gratings (0.35 or 1 μm deep) with/without Noc treatment (0.04 or 1 μm). *Yellow arrow* the long axis of FN line pattern or each micrograting. *Bar* 50 μm. **b** Average cell aspect ratio (R) of cells on flat, FN-lines, or 1 μm gratings (0.35 or 1 μm deep) with/without Noc treatment. *n* number of cells. **P < 0.01 and ***P < 0.001. Data were analyzed using one-way ANOVA and a Bonferroni post hoc test. *Error bar* denotes the standard deviation of the mean

Since cell alignments and elongation on 1 µm deep gratings are not inhibited by the depletion of actin filaments, we implied that MTs play more important roles in determining both alignments and length as compared to actin filaments. To verify the role of MTs on cell alignment and elongation in 1 µm deep gratings, we treated cells with Noc at 0.04 or 1 μm immediately after they were located on each surface. It was reported that at 0.04 μm, Noc altered MT dynamics but did not alter the disassembly of net MTs. However, at concentrations higher than 0.04 μm, the disassembly of net MTs was altered [22]. We also observed this concentration-dependent effect of Noc on MTs of cells in all the substrates. Furthermore, MTs were depleted not at 0.04 μm but at 1 μm of Noc (Fig. 5). At 0.04 µm of Noc, there was misalignment of the cells on flat surfaces, FN-lines, and 0.35 µm deep gratings when compared with the untreated cells on the same substrates (Fig. 6a; Table 2). In fact, R values of treated cells on these three substrates were significantly lower than those of untreated cells on the same substrates (Fig. 6b; Table 3). However, cell alignment and cell elongation on 1 µm deep gratings were not significantly affected by Noc at a concentration of 0.04 µm. However, at 1 µm concentration of Noc, there was misalignment (Fig. 6a; Table 2) of cells and a decrease in R values (Fig. 6b; Table 3). These results indicate that cell alignment and elongation on both FN-lines and 0.35 µm deep gratings were easily interfered only by altering MTs dynamics, while those on 1 µm deep gratings were interfered when net MTs were disassembled. This is in line with the recently proposed control mechanism that is dependent on MTs. These results also suggest that both actin filaments and MTs are instrumental in the alignment and elongation of cells, which are induced by either chemical pattern or 0.35 µm deep gratings. However, MTs alone could accomplish the alignment and elongation of cells on 1 µm deep gratings.

## Conclusions

In this study, we investigated how chemical and physical anisotropies on the surface affected cell alignment and elongation in RPE-1 cells using μCP and microfabrication. Moreover, we also verified that cell elongation strongly depends on the depth of microgratings. Previously, the relative importance of actin filaments and MTs in response to the anisotropic structures has not been determined. Our results indicate that neither cortical actin filament nor FA is a prerequisite for alignment and elongation of cells on 1 µm gratings (1 µm depth) and 2 ratings (2 μm depth). Furthermore, MTs dynamics were imposed by geometrical constraints, such as depth. These parameters played an essential role in the alignment and elongation of non-transformed RPE-1 cells.

The influence of microgratings on cell elongation and alignment is routinely observed in many different cell types including normal fibroblast cells. However, these microgratings may have a different effect on cancer epithelial cells that have impaired MT stabilities. Thus, these studies with non-transformed epithelial cells would determine whether deregulated behaviors can contribute to carcinogenesis. In addition to our basic understanding of cellular response and anisotropic surface structures, our findings provide hints for the application of synthetic structures, which are used for the differentiation of stem cells. Micro- and nanogratings are known to enhance neural differentiation of stem cells through cell elongation [23, 24].

## Experimental sections

### Preparation of FN-line pattern and microgratings

1 μm gratings (1 μm ridge width, 2 μm pitch) having two different groove depths (0.35 or 1 μm); these gratings were developed using PDMS (polydimethylsiloxane) (Sylgard® 184, Dow Corning) by soft lithography [25]. In a 4-inch silicon wafer, we fabricated master molds with grating structures using standard photolithography. After successfully conducting the fabrication process, we performed a reactive ion etching process. For this purpose, 10 mL of PDMS and its curing agent were mixed in a 10:1 ratio (w/w). The resultant mixture was then poured into each mold and cured at 65 ℃ for 2 h. To improve cell adhesion on the grating structure, the gratings were coated with 2 µg/mL solution of FN (Sigma Chemical. Co.) at 37 °C for 1 h.

The FN-line pattern was prepared on TCPS by microcontact printing (μCP) [9]. In this process, a PDMS stamp with gratings (1 μm ridge width, 2 μm pitch, 1 μm depth) was immersed for 1 h into a solution containing Cy3.5-labeled FN (final conc. 0.2 mg/mL) (GE Healthcare) and bare FN (final conc.16 µg/mL). Then, the stamp was air-dried for 30 min at room temperature before printing FN onto TCPS. In the meantime, the TCPS surface was oxidized in a plasma chamber (Harrick Plasma, Ithaca, NY, USA) for 3 min. The PDMS stamp was carefully placed onto a freshly cleaned TCPS for 30 min. As soon as the stamp was removed from the TCPS surface, we poured 0.2 % Pluronic F-127 (Sigma Chemical Co.) in phosphate-buffered saline (PBS) into TCPS. Then, we incubated it for 1 h at room temperature to make the uncoated area passive. Before cell seeding, TCPS was washed twice with PBS.

### Cell culture

RPE-1 (ATCC #CRL-4000) cells were maintained in Dulbecco’s Modified Eagle Medium (DMEM) (Thermo Fisher Scientific, Pittsburgh, PA, USA), which was supplemented with 10 % fetal bovine serum (FBS). Stock cell cultures were maintained in a humidified incubator at 37 °C in an atmosphere with 5 % CO_2_.

### Drug treatments

We added cytochalasin D (CD) (0.1–1 μm), actin polymerization inhibitor, or nocodazole (Noc) (0.04–1 μm), a MT polymerization inhibitor, right after cell seeding. CD and Noc were purchased from Sigma Chemical Co.

### Immunofluorescence microscopy

For indirect immunofluorescence microscopy, cover glass cultures were washed with PBS, fixed with 4 % paraformaldehyde (Electron Microscopy Sciences, Hatfield, PA, USA) and permeabilized with 0.5 % Triton X-100 in PBS as previously detailed [26]. In this process, mouse anti-α-tubulin (Cell Signaling Technology, Seoul, Korea) and mouse anti-vinculin (Sigma Chemical Co.) were used as primary antibody (1:1000 dilution), while fluorescein isothiocyanate (FITC)-conjugated anti-mouse (Sigma Chemical Co.) was used as the secondary antibody (1:5000 dilution). For F-actin staining, cells were incubated with 5 µg/mL of tetramethyl rhodamine B isothiocyanate (TRITC)-phalloidin (Sigma Chemical Co.) in PBS for 30 min. Image stacks were acquired and de-convoluted using a Delta Vision System (GE Healthcare) centered on an IX70 inverted microscope (Olympus), which was equipped with a CoolSNAP HQ2 charge-coupled device camera (Photometrics, Tucson, AZ, USA).

### Live cell microscopy and quantification

For live-cell imaging, cells were placed on either flat surface of TCPS, FN line pattern, or 1 µm gratings (0.35 or 1 µm deep) and incubated for 1 h. Time-lapse images were captured every 5 min for 24 h at 37 °C using Biostation CT (Nikon). Time-lapse image sequences were compiled and measured using Image J (NIH, USA). The cell aspect ratio (R) was determined by measuring cell length (L) and cell width (W) at 12 h after seeding. In this case, L is defined as the length of the long axis of the cell, while W is defined as the maximum width of a cell axis that is perpendicular to the long axis [27]. Cell alignment is defined as the angular difference between the major axis of cells and the long axis of FN-line or each micrograting and was measured at 12 h after seeding with and without CD or Noc treatment. Cells with alignment angle less than 15° are deemed to be aligned [9].

### Statistical analysis

Every experiment was conducted twice or three times on different days. All the data were expressed as mean ± SD. Data were analyzed using one-way ANOVA and a Bonferroni post hoc test. Statistical analyses were performed using GraphPad Prism 5.0 (GraphPad Software Inc., La Jolla, CA) and a two-tailed hypothesis. *P* values <0.01 were considered to be statistically significant.

## Additional file

10.1186/s12951-016-0187-8 (A) A phase contrast image of TCPS surface. Bar, 100 μm. (B) An imageshowing FN-lines (1 μm line and spacing) obtained by Atomic Force Microscopy (AFM) (Dimension 3100with a Nanoscope III controller, Digital Instruments) using silicon cantilevers (spring constant; 50 Nm^-1^)(RTESP, Veeco Probes) in contact mode. (C-E) SEM (Scanning electron microscopy) (6010 LV, JEOL)images showing the cross section of three different microgratings; 1 μm gratings with 0.35 um depth (C) and1 μm depth (D) and 2 μm gratings with 2 μm depth (E). **Figure S2.** (A) Fluorescence image of a RPE-1 cell stably expressing GFP/centrin cell on 1 μm gratings (1 μm deep). Bar, 30 μm. A yellow arrow indicates the direction of cell elongation. (B) Average cell aspect ratio (R) of cells on 1 μm gratings (0.35 or 1 μm deep) and 2 μm gratings with/without CD treatment. n: number of cells. ****P* < 0.001. Data were analyzed using one-way ANOVA and a Bonferroni *post hoc* test. Error bar denotes the standard deviation of the mean. **Figure S3.** Alignment of actin and vinculin to the different substrates (Flat TCPS surface, FN-lines, and 1 μm gratings (0.35 or 1μm deep)). The alignment angle was measured as an angle difference of actin or vinculin orientation to the long axis of a cell on flat PDMS surface or the long axis of the FN-line or each micrograting. #: the number of cells. Error bar denotes the standard deviation of the mean. **Figure S4.** Merged image of MTs (Green fluorescence) and pattern (phase contrast) of cells on 1 μm grating (1 μm deep) in the presenceof CD at 1 μM.

## References

[1] Curtis A, Wilkinson C (1997). Topographical control of cells. Biomaterials.

[2] Hubbell JA (2003). Materials as morphogenetic guides in tissue engineering. Curr Opin Biotechnol.

[3] Juliano RL, Haskill S (1993). Signal transduction from the extracellular matrix. J Cell Biol.

[4] Lim JY, Donahue HJ (2007). Cell sensing and response to micro- and nanostructured surfaces produced by chemical and topographic patterning. Tissue Eng.

[5] Flemming RG, Murphy CJ, Abrams GA, Goodman SL, Nealey PF (1999). Effects of synthetic micro- and nano-structured surfaces on cell behavior. Biomaterials.

[6] Oakley C, Jaeger NA, Brunette DM (1997). Sensitivity of fibroblasts and their cytoskeletons to substratum topographies: topographic guidance and topographic compensation by micromachined grooves of different dimensions. Exp Cell Res.

[7] Clark P, Connolly P, Curtis AS, Dow JA, Wilkinson CD (1987). Topographical control of cell behaviour. I. Simple step cues. Development.

[8] Crouch AS, Miller D, Luebke KJ, Hu W (2009). Correlation of anisotropic cell behaviors with topographic aspect ratio. Biomaterials.

[9] Wong ST, Teo SK, Park S, Chiam KH, Yim EK (2014). Anisotropic rigidity sensing on grating topography directs human mesenchymal stem cell elongation. Biomech Model Mechanobiol.

[10] den Braber ET, de Ruijter JE, Ginsel LA, von Recum AF, Jansen JA (1998). Orientation of ECM protein deposition, fibroblast cytoskeleton, and attachment complex components on silicone microgrooved surfaces. J Biomed Mater Res.

[11] Walboomers XF, Croes HJ, Ginsel LA, Jansen JA (1998). Growth behavior of fibroblasts on microgrooved polystyrene. Biomaterials.

[12] Foethke D, Makushok T, Brunner D, Nedelec F (2009). Force- and length-dependent catastrophe activities explain interphase microtubule organization in fission yeast. Mol Syst Biol.

[13] Levina EM, Kharitonova MA, Rovensky YA, Vasiliev JM (2001). Cytoskeletal control of fibroblast length: experiments with linear strips of substrate. J Cell Sci.

[14] Dunn GA, Heath JP (1976). A new hypothesis of contact guidance in tissue cells. Exp Cell Res.

[15] Domnina LV, Rovensky JA, Vasiliev JM, Gelfand IM (1985). Effect of microtubule-destroying drugs on the spreading and shape of cultured epithelial cells. J Cell Sci.

[16] Gauthier NC, Rossier OM, Mathur A, Hone JC, Sheetz MP (2009). Plasma membrane area increases with spread area by exocytosis of a GPI-anchored protein compartment. Mol Biol Cell.

[17] Théry M, Racine V, Piel M, Pépin A, Dimitrov A (2006). Anisotropy of cell adhesive microenvironment governs cell internal organization and orientation of polarity. Proc Nat Acad Sci USA.

[18] Mata J, Nurse P (1997). tea1 and the microtubular cytoskeleton are important for generating global spatial order within the fission yeast cell. Cell.

[19] Beroukim R, Mermel CH, Porter D, Wei G, Raychaudhuri S (2010). The landscape of somatic copy-number alteration across human cancer. Nature.

[20] Thery M, Pepin A, Dressaire E, Chen Y, Bornens M (2006). Cell distribution of stress fibres in response to the geometry of the adhesive environment. Cell Motil Cytoskeleton.

[21] Huda S, Soh S, Pilans D, Byrska-Bishop M, Kim J (2012). Microtubule guidance tested through controlled cell geometry. J Cell Sci.

[22] Vasquez RJ, Howell B, Yvon AM, Wadsworth P, Cassimeris L (1997). Nanomolar concentrations of nocodazole alter microtubule dynamic instability in vivo and in vitro. Mol Biol Cell.

[23] Teo BK, Wong ST, Lim CK, Kung TY, Yap CH (2013). Nanotopography modulates mechanotransduction of stem cells and induces differentiation through focal adhesion kinase. ACS Nano.

[24] Seo CH, Jeong H, Furukawa KS, Suzuki Y, Ushida T (2013). The switching of focal adhesion maturation sites and actin filament activation for MSCs. Biomaterials.

[25] Kim EH, Oh N, Jun M, Ko K, Park S (2015). Effect of cyclic stretching on cell shape and division. Biochip J.

[26] Lee K, Song K (2007). Actin dysfunction activates ERK1/2 and delays entry into mitosis in mammalian cells. Cell Cycle.

[27] Picone R, Ren X, Ivanovitch KD, Clarke JD, McKendry RA (2010). A polarised population of dynamic microtubules mediates homeostatic length control in animal cells. PLoS Biol.

