# Supplementary material for: Contribution of actin filaments and microtubules to cell elongation and alignment depends on the grating depth of microgratings
Source: J Nanobiotechnology. 2016 Apr 29;14:35. doi: 10.1186/s12951-016-0187-8 (PMC4850729; doi:10.1186/s12951-016-0187-8)
Supplement: Supplementary file 1 — 10.1186/s12951-016-0187-8 (A) A phase contrast image of TCPS surface. Bar, 100 μm. (B) An imageshowing FN-lines (1 μm line and spacing) obtained by Atomic Force Microscopy (AFM) (Dimension 3100with a Nanoscope III controller, Digital Instruments) using silicon cantilevers (spring constant; 50 Nm-1)(RTESP, Veeco Probes) in contact mode. (C-E) SEM (Scanning electron microscopy) (6010 LV, JEOL)images showing the cross section of three different microgratings; 1 μm gratings with 0.35 um depth (C) and1 μm depth (D) and 2 μm gratings with 2 μm depth (E). Figure S2. (A) Fluorescence image of a RPE-1 cell stably expressing GFP/centrin cell on 1 μm gratings (1 μm deep). Bar, 30 μm. A yellow arrow indicates the direction of cell elongation. (B) Average cell aspect ratio (R) of cells on 1 μm gratings (0.35 or 1 μm deep) and 2 μm gratings with/without CD treatment. n: number of cells. ***P < 0.001. Data were analyzed using one-way ANOVA and a Bonferroni post hoc test. Error bar denotes the standard deviation of the mean. Figure S3. Alignment of actin and vinculin to the different substrates (Flat TCPS surface, FN-lines, and 1 μm gratings (0.35 or 1μm deep)). The alignment angle was measured as an angle difference of actin or vinculin orientation to the long axis of a cell on flat PDMS surface or the long axis of the FN-line or each micrograting. #: the number of cells. Error bar denotes the standard deviation of the mean. Figure S4. Merged image of MTs (Green fluorescence) and pattern (phase contrast) of cells on 1 μm grating (1 μm deep) in the presenceof CD at 1 μM. [file 12951_2016_187_MOESM1_ESM.docx]

**Supporting information**

**Contribution of actin filaments and microtubules to cell elongation and alignment depends on the grating depth of microgratings**

Kyunghee Lee^1^, Ee Hyun Kim^1, 2^ Naeun Oh^2^, Nguyen Anh Tuan^1^, Nam Ho Bae^3^, Seok Jae Lee^3^, Kyoung G. Lee^3^, Chi-Yong Eom^4^, Evelyn K. Yim^1^ and Sungsu Park^5*^

^1^Mechanobiology Institute (MBI), National University of Singapore, Singapore 117411, Singapore

^2^Department of Chemistry and Nano Sciences (BK21 plus), Ewha Womans University, Seoul 120-750, Korea

^3^Department of Nano Bio Research, National Nanofab Center (NNFC), Daejeon 305-806, Korea

^4^Seoul Center, Korea Basic Science Institute, Seoul, 136-713, Korea

^5^School of Mechanical Engineering, Sungkyunkwan University, Suwon 440-746, Korea

^*^**Corresponding author:** S. Park

School of Mechanical Engineering, Sungkyunkwan University, Suwon 440-746, Korea

Tel: +82-31-290-7431/Fax: +82-31-290-5889/E-mail: nanopark@skku.edu

**Co-author email addresses**:

Kyunghee Lee: khee0326@hotmail.com. Ee Hyun Kim: nbhyuny@gmail.com. Naeun Oh: naeunn90@gmail.com. Nguyen Anh Tuan: mbinat@nus.edu.sg. Nam Ho Bae: nhbae@nnfc.re.kr. Seok Jae Lee: sjlee@nnfc.re.kr Kyoung G. Lee: kglee@nnfc.re.kr. Chi-Yong Eom: cyeom@kbsi.re.kr. Evelyn K. Yim: [eyim@nus.edu.sg](mailto:eyim@nus.edu.sg).

**
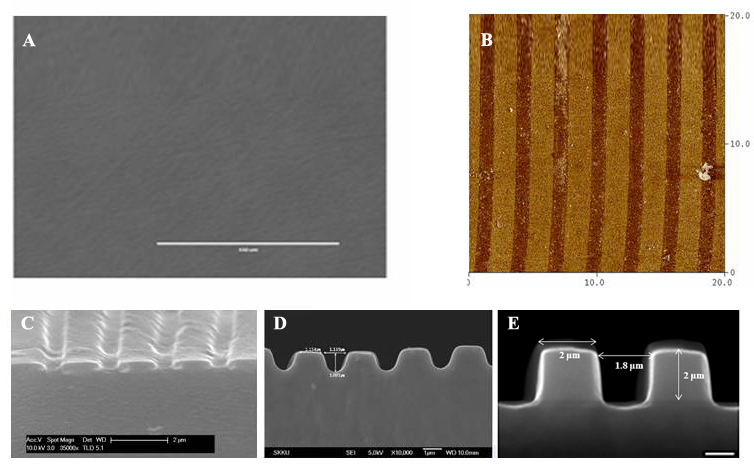
**

**Fig S1.** (A) A phase contrast image of TCPS surface. Bar, 100 μm. (B) An image showing FN-lines (1 μm line and spacing) obtained by Atomic Force Microscopy (AFM) (Dimension 3100 with a Nanoscope III controller, Digital Instruments) using silicon cantilevers (spring constant; 50 Nm^-1^) (RTESP, Veeco Probes) in contact mode. (C-E) SEM (Scanning electron microscopy) **(**6010 LV, JEOL) images showing the cross section of three different microgratings; 1 μm gratings with 0.35 um depth (C) and 1 μm depth (D) and 2 μm gratings with 2 μm depth (E).


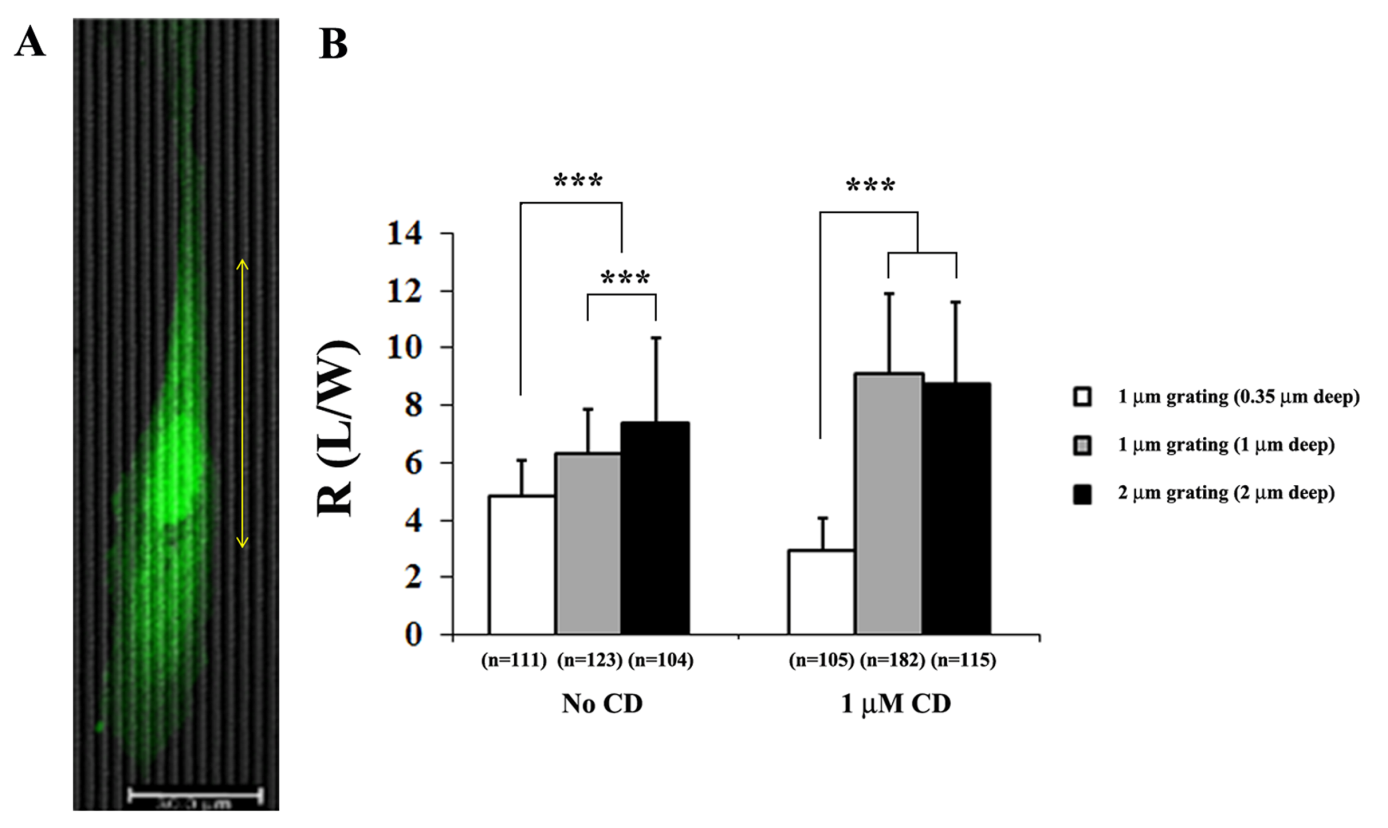


**Fig S2.** (A) Fluorescence image of a RPE-1 cell stably expressing GFP/centrin cell on 1 μm gratings (1 μm deep). Bar, 30 μm. A yellow arrow indicates the direction of cell elongation. (B) Average cell aspect ratio (R) of cells on 1 μm gratings (0.35 or 1 μm deep) and 2 μm gratings with/without CD treatment. n: number of cells. ****P* < 0.001. Data were analyzed using one-way ANOVA and a Bonferroni *post hoc* test. Error bar denotes the standard deviation of the mean.

**
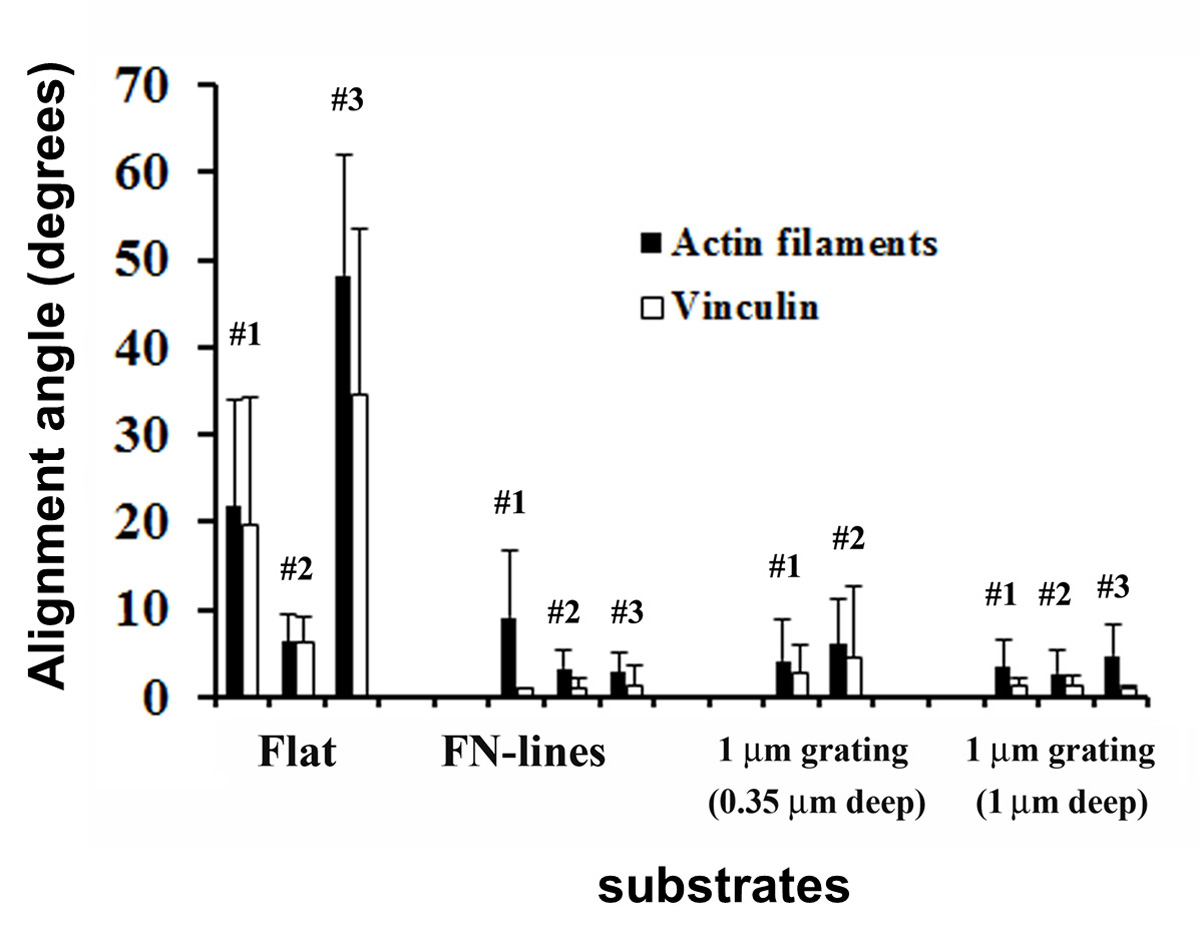
**

**Fig S3.** Alignment of actin and vinculin to the different substrates (Flat TCPS surface, FN-lines, and 1 μm gratings (0.35 or 1μm deep)). The alignment angle was measured as an angle difference of actin or vinculin orientation to the long axis of a cell on flat PDMS surface or the long axis of the FN-line or each micrograting. #: the number of cells. Error bar denotes the standard deviation of the mean.


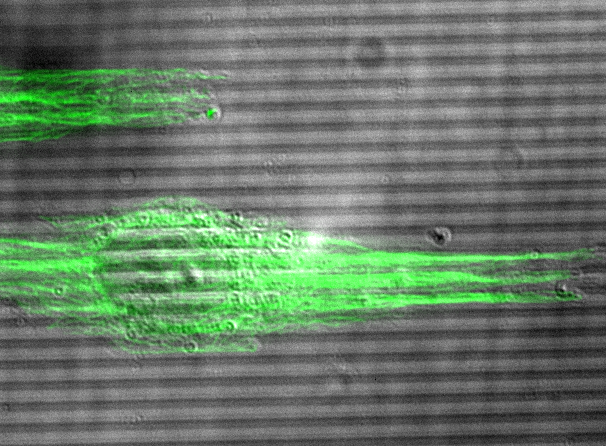


**Fig S4.** Merged image of MTs (Green fluorescence) and pattern (phase contrast) of cells on 1 μm grating (1 μm deep) in the presenceof CD at 1 μM.
